# Supplementary figures and images for: Silicon as a Functional Meat Ingredient Improves Jejunal and Hepatic Cholesterol Homeostasis in a Late-Stage Type 2 Diabetes Mellitus Rat Model
Source: Foods. 2024 Jun 7;13(12):1794. doi: 10.3390/foods13121794 (PMC11203255; doi:10.3390/foods13121794)

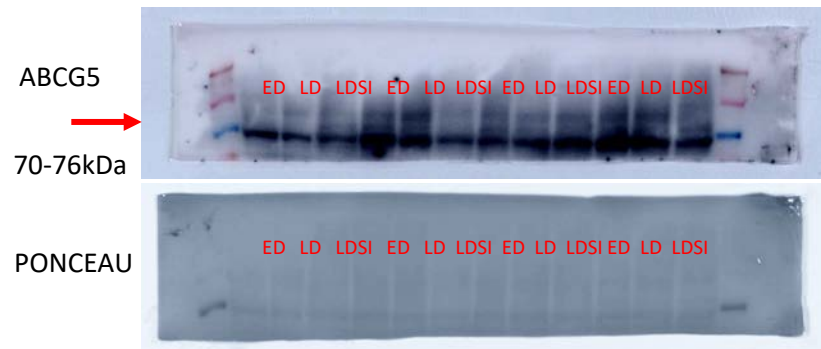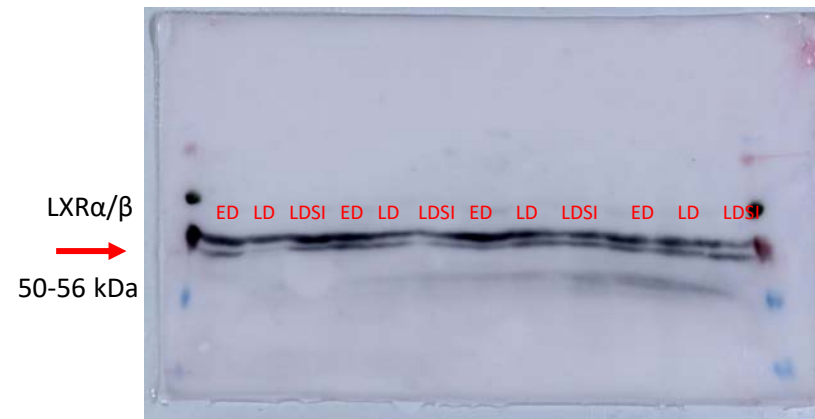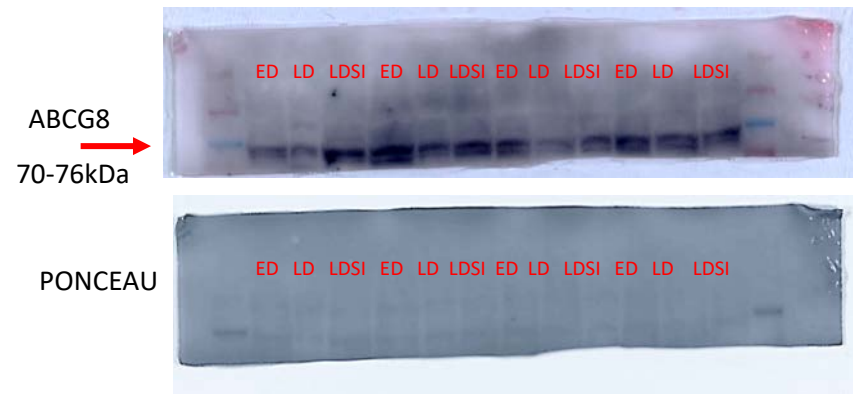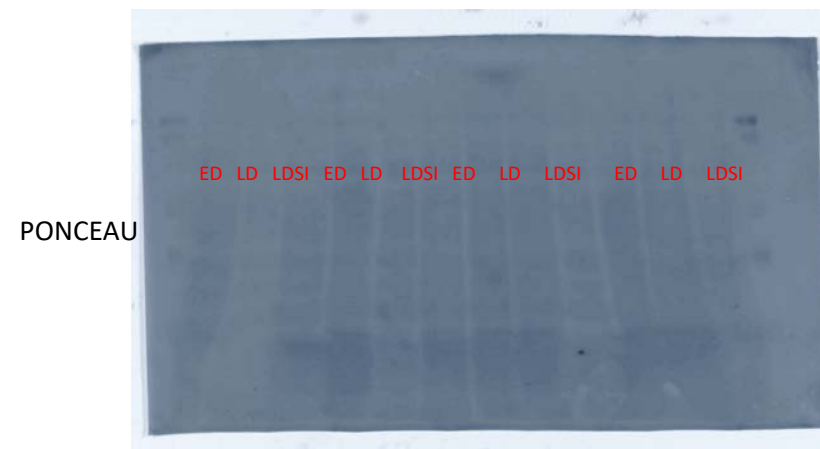

MTP  
→  
58kDa

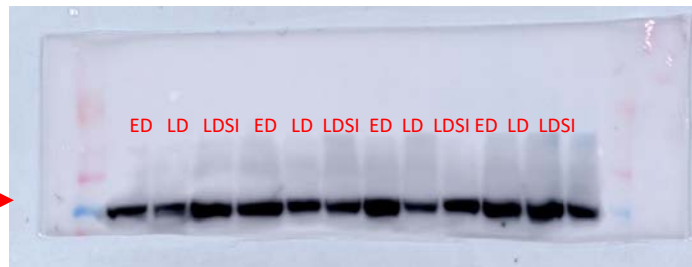

ACAT2  
→  
46kDa

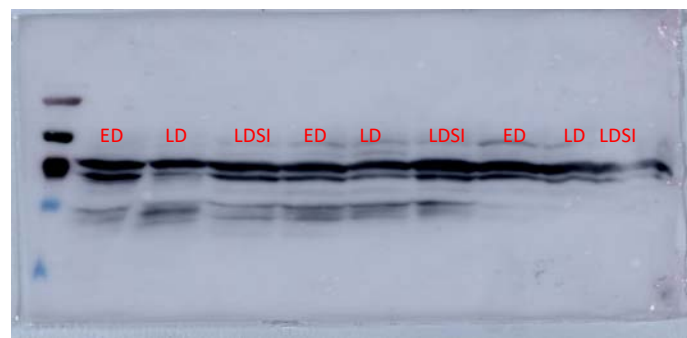

ACAT2  
→  
46kDa

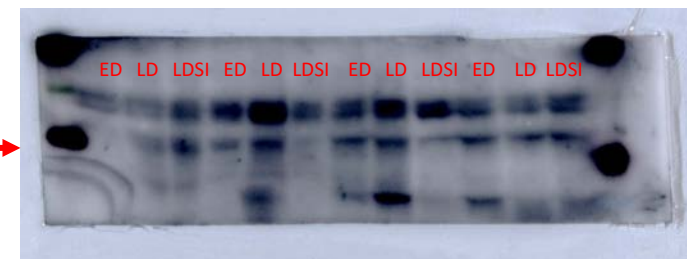

PONCEAU

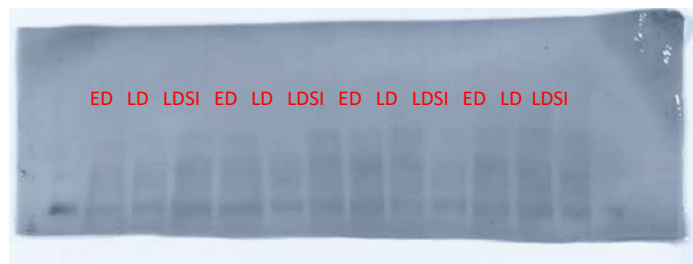

PONCEAU

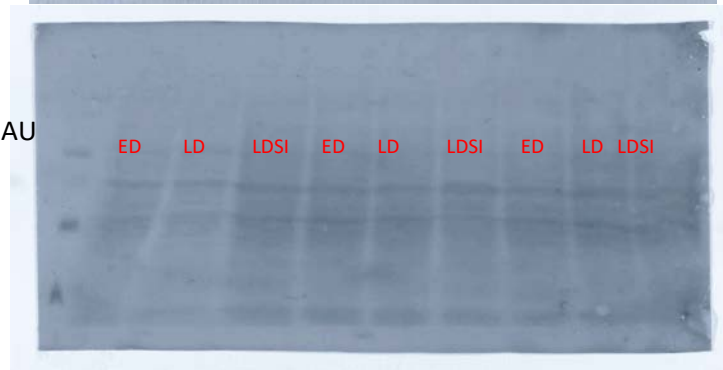

PONCEAU

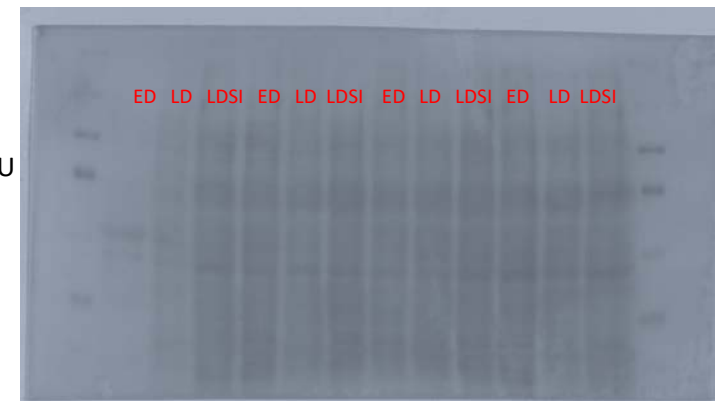

Supplement: Supplementary file 1 [file foods-13-01794-s001.zip › foods-3028811-supplementary.pdf]
